# Supplementary material for: Knowledge, Attitudes, and Acceptance of COVID-19 Vaccines among Secondary School Pupils in Zambia: Implications for Future Educational and Sensitisation Programmes
Source: Vaccines (Basel). 2022 Dec 14;10(12):2141. doi: 10.3390/vaccines10122141 (PMC9784903; doi:10.3390/vaccines10122141)
Supplement: Supplementary file 1 [file vaccines-10-02141-s001.zip › vaccines-2085945-supplementary.pdf]

Knowledge, attitude, and acceptance of COVID-19 vaccines among secondary school pupils in Zambia: implications for future educational and sensitisation programmes

Part I: Sociodemographic characteristics of pupils		
SN	Questions	Answers/choice
1	Age	years
2	Grade	
3	School	
4	Gender	1. Male<br>2. Female
5	Where do you live?	
6	Whom do you live with?	1. Parents<br>2. Guardians
Part II: Knowledge questions about COVID-19 vaccine [circle the correct answer]		

1	Have you ever heard about the COVID-19 vaccine?	1. Yes<br>2.No
2	If said "yes "to question 1 above, what is the main source of information?	1. Healthcare workers<br>2. TV/radio<br>3. Social media<br>4. Family/friends<br>5. Others.....
3	Do you know currently people taking the COVID-19 vaccine?	1. Yes 2.No 3. I don't know
4	Do you think COVID-19 vaccines reduce disease transmission?	1. Yes 2.No 3. I don't know
5	Do you think the use of COVID-19 vaccines has side effects?	1. Yes 2.No 3. I don't know
Part III: Attitude questions about the COVID-19 vaccine		

1	Taking the COVID-19 vaccine is important for our health.	1. Yes 2.No 3. I don't know
2	I will take the COVID-19 vaccine without any fear.	1. Yes 2.No 3. I don't know
3	I will encourage my family /friends to take the COVID-19 vaccine.	1. Yes 2.No 3. I don't know
4	COVID-19 vaccine should be distributed fairly to all of us.	1. Yes 2.No 3. I don't know
5	I support currently recommended COVID-19 vaccine campaigns and programs.	1. Yes 2.No 3. I don't know
Part IV: Factors influencing participants' acceptance of COVID-19 vaccines		
1	Have you ever suffered from COVID-19?	1. Yes 2.No 3. I don't know

2	Did any of your friends or relatives suffer from COVID-19?	1. Yes 2.No 3. I don't know
3	Has any of your friends or relatives died from COVID-19?	1. Yes 2.No 3. I don't know
4	Were you ever quarantined as a result of COVID-19?	1. Yes 2.No 3. I don't know
5	Are you able to practice physical and social distancing?	1. Yes 2.No 3. I don't know
6	Are the preventive measures of COVID-19 stressful to follow?	1. Yes 2.No 3. I don't know
7	Do you suffer from ANY chronic condition (tuberculosis, diabetes mellitus, HIV/AIDs, asthma, bronchitis, hypertension, cancer)?	1. Yes 2.No 3. I don't know

	Acceptance of the COVID-19 vaccine	
	Would you accept being vaccinated against COVID-19?	1. Yes 2.No 3. I don't know

Thank you for your participation!
